# Letter from the Editor in Chief

**DOI:** 10.19102/icrm.2022.13117

**Published:** 2022-11-15

**Authors:** Moussa Mansour


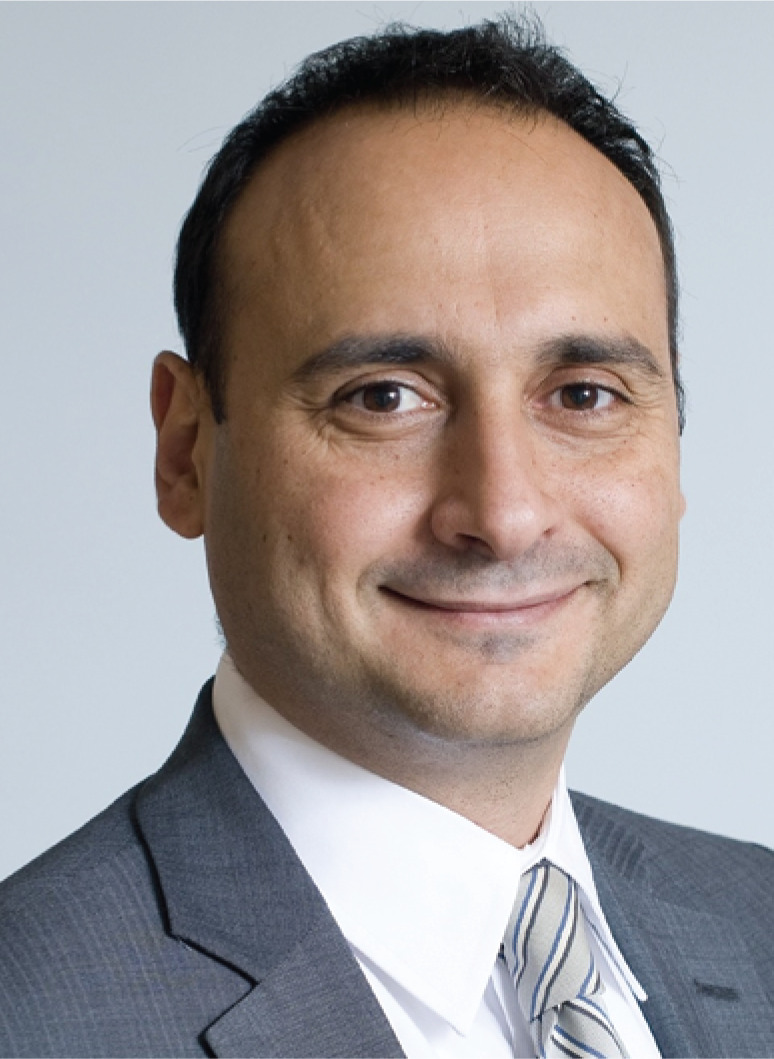


Dear readers,

The field of ambulatory cardiac monitoring has grown rapidly in the last few years. In particular, many studies have demonstrated the benefit of implantable loop monitors (ILRs) to detect previously undiagnosed atrial fibrillation (AF) in patients with cryptogenic stroke. The CRYSTAL AF study^1^ reported that ILRs detected AF in 12% of patients within 6 months of cryptogenic stroke. The PER DIEM study^2^ found that ILRs are superior to prolonged external monitoring for 30 days in detecting AF among patients with ischemic stroke and no prior evidence of the arrhythmia. In the LOOP study,^3^ ILR screening increased AF detection by 3-fold compared to usual care in individuals with stroke risk factors.

These and other studies have led to greater use of ILRs for AF detection. Technical advances in ILRs have also rendered them more effective in detecting arrhythmias. One such new feature is remote programming, which facilitates the use of ILRs and improves their efficiency, as described in the study by Mahajan et al.^4^ published in this issue of *The Journal of Innovations in Cardiac Rhythm Management*, which I encourage you to read. We have also been witnessing a rapid expansion in the use of consumer devices for the detection of cardiac arrhythmias. A recent consensus document from the AF-SCREEN International Collaboration^5^ provides a summary of the status of consumer-led screening for AF. However, more studies are needed to demonstrate the sensitivity and specificity of such an approach.

In summary, the positive results of outcome studies coupled with technological advances have catalyzed the rapid expansion of the field of ambulatory monitoring for cardiac arrhythmias, including specifically AF. Though most studies so far have used ILRs, consumer devices have been gaining popularity and may become the primary tool for arrhythmia diagnosis, especially if equipped with artificial intelligence algorithms that improve their specificity.

I hope that you enjoy reading this issue of *The Journal of Innovations in Cardiac Rhythm Management* and find its content educational. Best wishes to you and your families for a happy holiday season.


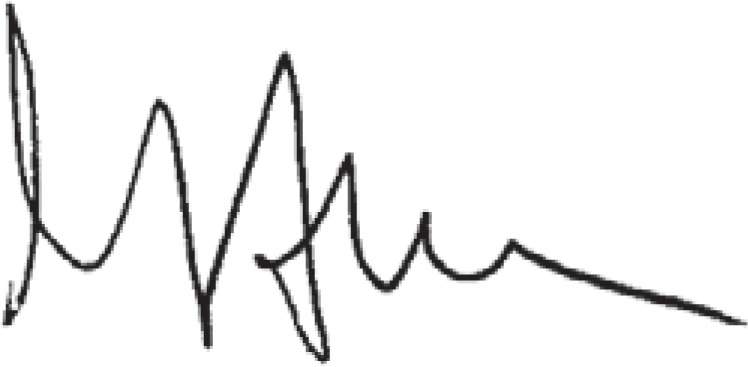


Sincerely,

Moussa Mansour, md, fhrs, facc

Editor in Chief

*The Journal of Innovations in Cardiac Rhythm Management*

MMansour@InnovationsInCRM.com

Director, Atrial Fibrillation Program

Jeremy Ruskin and Dan Starks Endowed Chair in Cardiology

Massachusetts General Hospital

Boston, MA 02114
